# From image to trust: Cross-national pathways to brand evangelism in hospitality sector

**DOI:** 10.1371/journal.pone.0350418

**Published:** 2026-05-28

**Authors:** Ngo Cao Nghia, Phan Khanh Duy, Bao Quoc Truong-Dinh

**Affiliations:** 1 Vietnam Aviation Academy, Ho Chi Minh City, Vietnam; 2 School of Management, Binh Duong University, Ho Chi Minh City, Vietnam; 3 University of Economics – The University of Danang‌‌, Danang City, Vietnam; Sichuan Agricultural University, CHINA

## Abstract

This study investigates how frontline employee attributes foster customer brand evangelism in ASEAN hospitality, addressing whether symbolic brand image or relational trust better explains advocacy and under what conditions these mechanisms vary across cultures. A two-wave survey design was implemented with 428 hotel guests in Vietnam and Thailand. Using PLS-SEM and multi-group analysis, the model tested digital competence, proactive assistance, and relationship-building as antecedents of brand evangelism, mediated by brand image and customer trust, and moderated by loyalty program participation and visit frequency. Results reveal dual pathways: brand image and trust both mediate employee behaviors, with symbolic cues dominating in Vietnam and relational credibility prevailing in Thailand. Loyalty programs strengthened trust-based evangelism in Thailand, while visit frequency shaped image-based evangelism differently across contexts. These mechanisms explained substantial variance in evangelism, underscoring cultural contingencies in advocacy formation. This research advances brand evangelism theory by integrating symbolic and relational mediators, identifying boundary conditions, and demonstrating cultural contrasts. It provides a novel dual-process, cross-national framework that refines relationship marketing and social identity perspectives while offering actionable guidance for culturally adaptive hospitality strategies.

## 1. Introduction

In the competitive environment of contemporary hospitality, organizations face the persistent challenge of sustaining differentiation in markets saturated with information and choice [1]. While traditional strategies of customer satisfaction and loyalty remain valuable, they are no longer sufficient to secure long-term competitive advantage. Increasingly, attention has shifted toward cultivating brand evangelists customers who move beyond passive advocacy to become passionate, proactive promoters of a brand [2,3]. Unlike conventional advocates, evangelists engage in zealous and voluntary efforts to persuade others, generating a ripple effect of influence that strengthens a brand’s visibility and legitimacy in the marketplace [4,5]. For hospitality firms, where frontline encounters strongly shape customer perceptions, understanding how such evangelism emerges is both a theoretical and practical imperative [6].

Recent scholarship has begun to clarify the antecedents of brand evangelism. [2] demonstrated that frontline employees’ digital competence, proactive assistance, and relationship-building significantly contribute to evangelism, operating indirectly through the mediating role of brand image, with the relationship further conditioned by perceived corporate social responsibility. While this framework represented a valuable advance, it remains constrained in three critical respects. First, the reliance on brand image as the sole mediating construct neglected the role of customer trust, a well-established driver of enduring relational outcomes in relationship marketing theory [7,8]. Second, the empirical scope was limited to Pakistan, raising questions of cross-cultural generalizability, particularly in hospitality contexts where service norms and consumer expectations are deeply embedded in national culture [9]. Third, the model did not incorporate boundary conditions such as loyalty program participation and visit frequency, factors that plausibly influence whether positive perceptions translate into evangelistic behavior [10].

These limitations lead to three interrelated research questions that guide the present study.

*RQ1: How do frontline employees’ digital competence, proactive assistance, and relationship-building foster customer brand evangelism across distinct national contexts?*

*RQ2: Through which mediating mechanisms specifically brand image and customer trust are these employees attributes translated into evangelism?*

*RQ3: Under what boundary conditions, such as loyalty program participation and visit frequency, are these mechanisms strengthened or weakened, and do these effects vary across cultural contexts such as Vietnam and Thailand?*

Addressing these questions matters for more than filling empirical gaps. By incorporating customer trust alongside brand image as dual mediators, the study refines relationship marketing theory by showing how perceptual and relational mechanisms jointly translate employee behaviors into evangelism. Introducing loyalty program participation and visit frequency as moderators extends social identity theory by identifying conditions under which brand alignment strengthens or weakens customer advocacy. Finally, by conducting a cross-national comparison between Vietnam and Thailand, two rapidly growing hospitality markets in ASEAN, the study advances theoretical understanding of whether these mechanisms are culturally invariant or contingent.

The contributions of this research are fourfold. First, it advances theory by integrating dual mediating mechanisms brand image and customer trust thereby offering a more complete explanation of how frontline employees’ competencies are transformed into evangelistic outcomes. Whereas prior work conceptualized brand image as the sole pathway, this study demonstrates that trust is equally central to the process, challenging outcome-centric models and extending relationship marketing theory with a dual-process account. Second, it extends social identity theory by identifying contextual moderators, showing that loyalty program participation and visit frequency act as boundary conditions under which identification with the brand translates into evangelism. This not only addresses calls for incorporating conditional variables but also provides a richer understanding of when and for whom evangelism is most likely to emerge. Third, the study broadens the empirical scope of brand evangelism research by conducting a cross-national comparison between Vietnam and Thailand, offering ASEAN-specific insights and testing the generalizability of the model beyond South Asia. This comparative design enables a theoretically meaningful assessment of whether evangelism is universally shaped by the same mechanisms or subject to cultural contingencies in service encounters. Finally, it contributes to practice by offering managers evidence-based strategies for leveraging digital competence, proactive service, and relationship-building to cultivate evangelism in ways that are sensitive to national culture and customer characteristics.

In so doing, the present research not only extends the empirical frontiers of brand evangelism but also refines its theoretical foundations, offering a framework that is both more comprehensive in its explanatory mechanisms and more sensitive to cultural and contextual variation.

## 2. Literature review

### 2.1. Brand evangelism in hospitality

Brand evangelism refers to customers’ zealous and voluntary promotion of a brand, extending beyond conventional advocacy to proactive persuasion of others [2,3]. Unlike advocacy, which reflects satisfaction and willingness to recommend, evangelism entails deeper emotional attachment and proactive behaviours that position customers as unpaid promoters [4]. While its significance for hospitality has been noted [5], prior studies often emphasise general antecedents such as satisfaction or loyalty, overlooking how frontline encounters generate such intense commitment. This gap is especially salient in Asian contexts, where cultural values like collectivism and relational harmony may amplify or constrain evangelism [9].

Existing literature is divided between symbolic and relational perspectives. Symbolic accounts highlight brand image, identity, and reputation as the main drivers [11,12], whereas relational perspectives stress trust, credibility, and enduring bonds [7,13]. Yet evangelism is both symbolic and relational: customers align with a brand’s image while assuming reputational risk, which demands trust. This study synthesises these perspectives by positioning brand image and trust as dual mediators, bridging theoretical divides and advancing relationship marketing and social identity theory.

ASEAN hospitality provides a fertile ground for this integration. Rapid digital transformation coexists with relational traditions, creating conditions to test symbolic versus relational mechanisms. Vietnam and Thailand exemplify hybrid consumption logics where modernisation pressures and collectivist norms intersect, offering insights that extend beyond cultural variation to inform how global hotel groups cultivate advocacy in emerging markets.

### 2.2. Theoretical foundations

The study of evangelism in hospitality has drawn from both social identity theory (SIT) and relationship marketing theory (RMT). SIT emphasizes that individuals derive part of their self-concept from group membership, which in the hospitality setting translates into customer identification with a brand through their encounters with frontline employees [14]. Prior research has shown that frontline employees’ behaviors whether digitally competent, proactive, or relational can foster this identification [15]. Yet, while SIT highlights the symbolic importance of brand alignment, it does not fully account for the relational mechanisms that sustain long-term evangelism. RMT provides that complement by emphasizing trust and relational quality as the foundation of advocacy and loyalty [7,8]. However, existing studies often treat these theories separately, with limited attempts to integrate symbolic identification and relational trust into a single explanatory framework. This theoretical fragmentation leaves unanswered the question of whether evangelism emerges more from symbolic brand fit or from trust in the relational processes underpinning service delivery.

To clarify how these theories map onto the study’s core variables: SIT grounds the brand image pathway, in which employees’ digital competence, proactive assistance, and relationship-building behaviors signal the hotel’s symbolic identity and social prestige. Customers who identify with this identity are motivated to publicly associate themselves with the brand a form of group affiliation which manifests as evangelistic behavior [14]. RMT, by contrast, grounds the customer trust pathway: consistent, credible frontline interactions cultivate belief in the brand’s reliability and benevolence. This relational assurance reduces the perceived risk of evangelism, enabling customers to advocate on the brand’s behalf even when personal reputational stakes are involved [7,8]. The two boundary conditions loyalty program participation and visit frequency extend both theories by specifying the structural and experiential contexts under which each pathway is amplified. Loyalty programs institutionalize SIT-driven identification, while repeated encounters test the cumulative trust posited by RMT.

### 2.3. Brand image as a mediator

Brand image has been widely recognized as a central mediator through which employee actions influence customer outcomes [11,12]. A favorable image consolidates perceptions of competence and service quality, providing customers with a symbolic anchor that strengthens their willingness to advocate for the brand [16]. In the study of [2], brand image played a pivotal role in linking frontline employees’ digital competence, proactive assistance, and relationship-building to evangelism. Yet this reliance on a single mediating construct risks oversimplifying a complex process. Brand image reflects an aggregated perception, but it does not capture whether customers believe those perceptions are trustworthy or authentic. Thus, while brand image provides valuable symbolic capital, it may not be sufficient to explain why customers progress from favorable evaluations to zealous promotion. The overemphasis on brand image in prior models risks obscuring other relational mechanisms that could provide a fuller account of evangelism.

### 2.4. Customer trust as a mediator

Trust is a cornerstone of relationship marketing theory, reflecting the belief that the brand and its representatives are reliable, competent, and motivated by customer well-being [7]. In hospitality, where encounters are high-stakes and emotionally charged, trust determines whether customers interpret employee behaviors as credible signals of the brand’s values. While prior work has examined trust as a predictor of loyalty and satisfaction [17], its role as a mediator between frontline behaviors and evangelism remains underexplored. This omission is theoretically significant. Trust explains why customers act on their positive evaluations, converting favorable perceptions into advocacy behaviors that involve reputational risk for the customer. Without incorporating trust, models of evangelism risk being incomplete, capturing only surface-level symbolic identification rather than the deeper relational security that motivates passionate promotion. Addressing this gap requires positioning trust alongside brand image to provide a dual-process account of how frontline employees influence evangelism.

### 2.5. Moderating conditions

Although prior research has highlighted the direct and mediated pathways to evangelism, limited attention has been given to contextual moderators that may condition these effects. Loyalty program participation represents a critical boundary condition, as such programs institutionalize the customer–brand relationship and provide external structures that reinforce identification and commitment. From the perspective of self-determination theory, membership may satisfy needs for competence (through privileged access), relatedness (through affiliation), and autonomy (through personalized rewards), thereby strengthening the link between brand image and evangelism. This aligns with customer–brand relationship frameworks, which suggest that formalized affiliation heightens symbolic identification and transforms image-based admiration into advocacy behaviors [10].

Similarly, visit frequency is likely to shape how trust translates into evangelism. Relationship marketing theory posits that trust develops cumulatively through repeated interactions, but self-determination theory highlights that repeated experiences can either sustain intrinsic motivation or undermine it if expectations are unmet. Repeat visitors may therefore require deeper relational reinforcement to maintain enthusiasm, while first-time guests may rely more heavily on immediate impressions that satisfy short-term relational or experiential needs. The absence of such moderators in prior research reflects a critical oversight, as it assumes homogeneity in customer responses and neglects the theoretical possibility that evangelism is contingent on relational depth, motivational fulfilment, and repeated engagement.

### 2.6. Cross-national perspective

Finally, the generalizability of existing models remains uncertain, as most studies including [2] are confined to single-country settings. This limitation is particularly salient given that hospitality experiences are deeply shaped by national culture. Vietnam and Thailand, for example, are both rapidly expanding hospitality markets, yet they differ in cultural norms, levels of digital adoption, and customer expectations of service encounters [18,19]. A cross-national design enables the assessment of whether the pathways linking frontline employee behaviors to evangelism are culturally invariant or contingent. Testing measurement invariance across these contexts is not simply a methodological requirement but a theoretical necessity, as it determines whether existing frameworks can claim universality or require cultural refinement. The neglect of cross-national comparison in prior work thus represents a substantial gap, limiting both the theoretical reach and practical utility of existing models. Table 1 provides a systematic positioning of the current study relative to key prior works, highlighting how the present study uniquely combines dual mediators, cross-national comparison, and moderating boundary conditions in a single integrated framework.

**Table 1:** Systematic literature review and study positioning.

Author(s), Year	Context	Key Constructs and Mechanisms	Methodology	Main Findings	Identified Gaps	Study Positions
Matzler et al. (2007)	Consumer brands (general)	Extraversion, brand passion, evangelism	Survey, SEM	Extraversion positively predicts evangelism	Focused on personality traits, not service encounters	Moves beyond individual traits to examine frontline employee behaviors as antecedents
Nguyen and Leblanc (2001)	Service industries	Corporate image, reputation, retention	Survey	Brand image central to retention decisions	Did not address evangelism or relational trust	Positions brand image as one mediator but integrates with trust to explain evangelism
Morgan and Hunt (1994)	B2B services	Commitment–trust framework	Conceptual, empirical tests	Trust as foundation of relationship marketing	Not linked to evangelism; no symbolic pathways	Extends commitment–trust theory into evangelism by adding dual mediators
Mansoor et al. (2025)	Pakistan, hospitality	Digital competence, proactive assistance, relationship-building → brand image → evangelism; CSR control	Survey, PLS-SEM	Employee attributes influence evangelism via brand image; CSR moderates	Single mediator (brand image); single-country; no moderators like loyalty/visit frequency	Extends with dual mediators (image + trust), introduces moderators, and tests cross-nationally (Vietnam/Thailand)
Shang and Li (2024)	Hospitality, ritualistic service	Service rituals, interaction quality, memory → evangelism	Survey, SEM	Ritualistic service enhances evangelism	Narrow focus on rituals; lacks digital competence and relational trust	Broadens scope to frontline competence, proactive service, and relationship-building
Sharma et al. (2022)	Brand communities, social media	Engagement, evangelistic tendencies	Survey	Online engagement drives evangelism	Focus on digital communities, not hospitality encounters	Extends to offline hospitality contexts with service encounters
Sohaib et al. (2022)	Green hotel brands	Nature-based solutions, well-being, attitude, loyalty, evangelism	Survey	Green initiatives influence evangelism	Focused on sustainability; did not test dual mediators or cross-national	Shifts lens from green branding to employee-driven mechanisms with cultural contingencies
Good and Schwepker (2022)	B2B sales	Political skill, relationship-building, performance	Survey	Relationship-building critical in B2B	Did not link relationship-building to evangelism	Directly tests relationship-building as antecedent of evangelism
Current Study (2025)	Hospitality, Vietnam and Thailand	Employee digital competence, proactive assistance, relationship-building → brand image and trust → evangelism; moderators: loyalty program and visit frequency; CSR control	Two-wave cross-national survey, PLS-SEM	Dual mediators explain symbolic (Vietnam: brand image) and relational (Thailand: trust) mechanisms; boundary conditions shape outcomes	Addresses gaps in mediation, moderation, and cross-national generalizability	Positions itself as a dual-process, cross-cultural refinement of relationship marketing and SIT, offering theoretical integration and ASEAN-specific insights

Source: Authors’ own work.

### 2.7. Hypotheses development

Frontline employees’ digital competence has been increasingly recognized as central to contemporary hospitality encounters. The ability to manage digital tools and platforms not only enables efficient service delivery but also enhances personalization, creating experiences that resonate with tech-savvy consumers [2]. Yet, while competence in digital service delivery has been shown to predict satisfaction, its potential to inspire evangelism remains underexplored. Customers may interpret digital proficiency as a signal of innovation and reliability, qualities that encourage them to actively recommend the brand. To validate this underexamined pathway, the following hypothesis is proposed:

*H1a: Frontline employees’ digital competence positively influences customers’ brand evangelism.*

Proactive assistance, defined as employees’ anticipatory engagement in solving customer needs before they arise, is another attribute critical for service differentiation [20]. While prior studies have linked proactive behaviors to satisfaction and loyalty, little empirical work has examined their direct role in motivating customers to become evangelists. Anticipatory care not only creates delight but also fosters a sense of personalized attention that customers may wish to share with others. Testing this link addresses the gap in understanding whether proactive service behaviors extend beyond satisfaction to stimulate zealous advocacy. Thus, it is hypothesized:

*H1b: Frontline employees’ proactive assistance positively influences customers’ brand evangelism.*

Relationship-building has long been identified as the foundation of long-term customer commitment [7]. In hospitality, where relational interactions dominate, building trustful and enduring ties is likely to inspire customers to promote the brand voluntarily. Yet, much of the literature has focused on relationship-building as a predictor of retention or loyalty rather than evangelism. Testing this pathway addresses whether emotional bonds cultivated in service encounters translate into advocacy that is passionate and proactive rather than merely transactional. Therefore, it is hypothesized:

*H1c: Frontline employees’ relationship-building positively influences customers’ brand evangelism.*

A strong brand image consolidates customers’ perceptions into a symbolic identity that can enhance willingness to advocate [11,12]. However, limited attention has been given to how employee attributes such as digital competence contribute to shaping this image. In an increasingly digital service environment, competent use of technology may signal modernity and reliability, strengthening the overall brand image. Testing this pathway clarifies the perceptual mechanisms through which digital competence translates into customer outcomes. Hence, the hypothesis is advanced:

*H2a: Digital competence positively influences brand image.*

Proactive assistance, by creating memorable and personalized experiences, is also likely to enhance perceptions of the brand. Customers who experience anticipatory service may form impressions of the brand as attentive, caring, and customer-oriented [21]. While prior studies highlight links between proactive behaviors and satisfaction, few have explicitly connected them to brand image formation. Testing this link offers a novel contribution by positioning proactive assistance as a driver of symbolic brand perceptions. Thus, it is hypothesized:

*H2b: Proactive assistance positively influences brand image.*

Similarly, relationship-building contributes to a favorable brand image by signaling that the organization values long-term ties rather than short-term transactions. Strong relational bonds elevate customers’ perception of the brand as trustworthy and relationally committed [4]. Yet this connection has rarely been tested in hospitality contexts, where relational service encounters are central. Addressing this gap justifies the following hypothesis:

*H2c: Relationship-building positively influences brand image.*

While brand image captures symbolic evaluations, trust reflects customers’ belief that the brand consistently acts in their interest. Digital competence may directly enhance trust by demonstrating reliability in managing service interactions. Customers are more likely to trust brands that can competently deploy technology to safeguard transactions and provide efficient solutions. Yet the trust-building function of digital competence remains underexplored in prior work. To address this omission, it is hypothesized:

*H3a: Digital competence positively influences customer trust.*

Proactive assistance also strengthens trust by signaling employee sincerity and concern for customer welfare [2]. Anticipating problems before they arise conveys credibility and authentic care, qualities that foster confidence in the brand. Testing this link is important for demonstrating whether proactive behaviors build not just satisfaction but also relational assurance. Accordingly, it is hypothesized:

*H3b: Proactive assistance positively influences customer trust.*

Relationship-building has traditionally been linked to trust development in relationship marketing theory [7]. Customers who perceive genuine relational investment are more likely to believe in the brand’s reliability and integrity. While this pathway seems intuitive, empirical work on evangelism has not fully tested how relationship-building translates into trust as a precursor to advocacy. Thus, the study advances the following hypothesis:

*H3c: Relationship-building positively influences customer trust.*

Downstream, both brand image and trust are expected to directly foster evangelism. A favorable brand image not only strengthens identification but also legitimizes customers’ willingness to advocate on behalf of the brand [12,22]. Without a strong image, evangelistic behaviors may lack credibility. Therefore, it is hypothesized:

*H4: Brand image positively influences customers’ brand evangelism.*

Customer trust, by contrast, provides the relational assurance that transforms positive evaluations into proactive behaviors. Because evangelism entails reputational risk, customers are unlikely to engage unless they trust the brand to maintain its promises and not jeopardize their credibility [15]. Testing this relationship fills a critical gap in explaining why admiration alone is insufficient to generate evangelism. Hence, it is hypothesized:

*H5: Customer trust positively influences customers’ brand evangelism.*

Building on these direct pathways, brand image and trust are expected to function as mediators that explain how employee behaviors are internalized into evangelistic outcomes. Brand image has consistently been identified as a critical symbolic mechanism that translates service encounters into perceptions of brand reputation and identity [11,12]. When customers perceive employees as digitally competent, proactive, and relationally attentive, they are more likely to form favorable brand images, which in turn inspire them to speak positively and advocate zealously for the brand. Prior studies have treated brand image as an endpoint, but its mediating role in linking frontline behaviors to evangelism has received less systematic attention across national contexts. Testing this pathway is theoretically important, as it clarifies whether evangelism stems primarily from symbolic admiration of the brand’s image rather than directly from service interactions. Accordingly, the study hypothesizes:

*H6a: Brand image mediates the relationship between frontline employees’ attributes and brand evangelism.*

Trust, however, offers a distinct relational mechanism that complements symbolic evaluations. Relationship marketing theory emphasizes that trust is foundational for long-term customer commitment [7]. Unlike brand image, which reflects how the brand is perceived, trust reflects the confidence customers place in the brand to consistently deliver on its promises. In the context of evangelism, where customers voluntarily take on reputational risk by recommending the brand, trust is particularly critical. Without trust, favorable perceptions may remain superficial and fail to translate into proactive advocacy. Yet, despite its theoretical centrality, trust has rarely been tested as a mediator in evangelism models, leaving a significant gap in understanding how relational security shapes customers’ willingness to evangelize. Addressing this omission, the study advances the following hypothesis:

*H6b: Customer trust mediates the relationship between frontline employees’ attributes and brand evangelism.*

The influence of image and trust on evangelism is unlikely to be uniform across customers, necessitating examination of moderating conditions. Loyalty program participation formalizes the customer–brand bond and may strengthen the symbolic translation of image into evangelism. However, prior studies have focused primarily on loyalty programs as retention mechanisms rather than as drivers of proactive advocacy [10]. Testing this interaction addresses an overlooked pathway in the literature. Thus, it is hypothesized:

*H7: Loyalty program participation moderates the relationship between brand image and brand evangelism, such that the effect is stronger for loyalty members.*

Visit frequency offers another boundary condition. While repeated encounters can reinforce trust through consistent experiences, they can also elevate expectations, which, if unmet, may erode enthusiasm [23]. This dual possibility has rarely been tested, with prior research often assuming linear positive effects. Testing frequency as a moderator clarifies whether trust translates into evangelism consistently or whether relational fatigue may weaken the effect. Hence, it is hypothesized:

*H8: Visit frequency moderates the relationship between customer trust and brand evangelism, such that the effect differs by level of visit frequency.*

**Fig 1:**
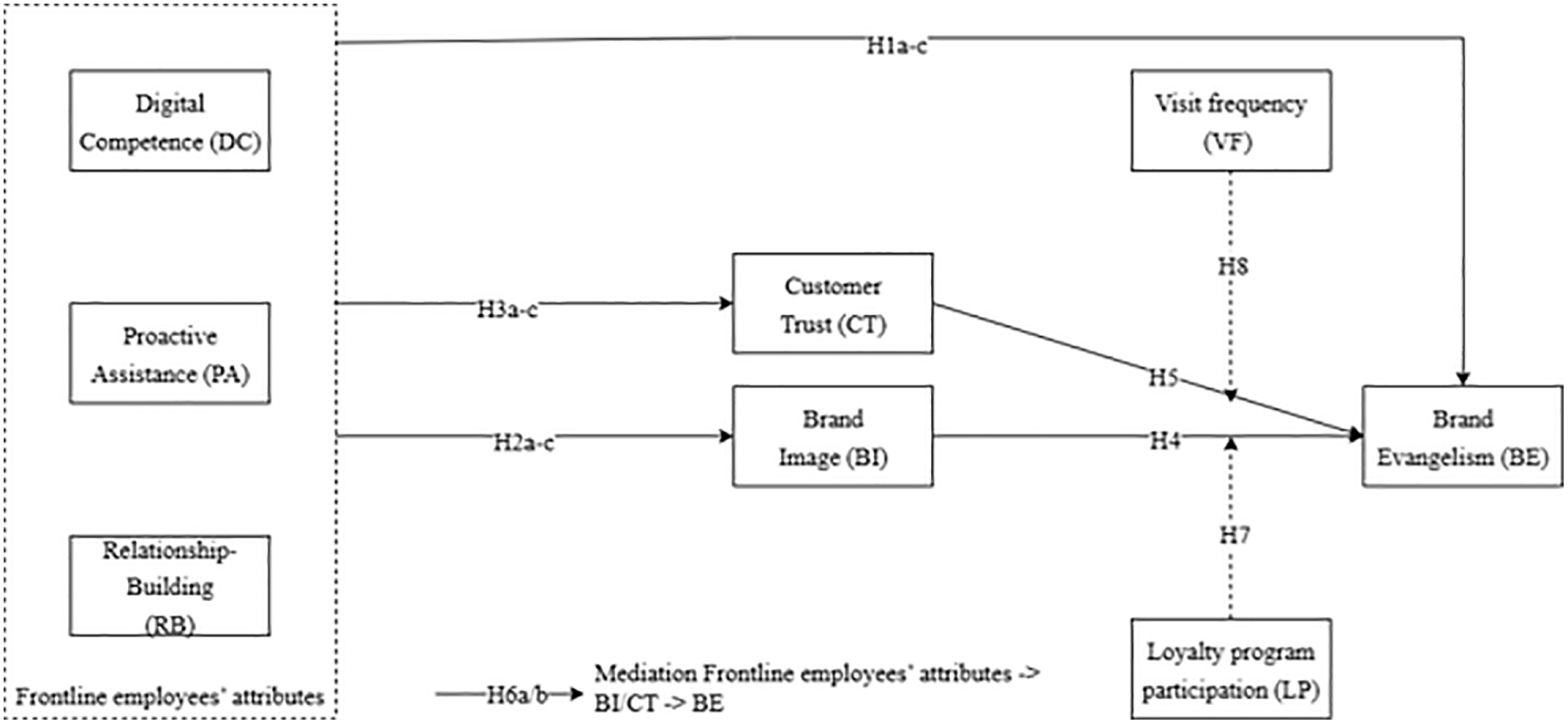
Proposed Research Model. The model depicts three frontline employee antecedents (digital competence, proactive assistance, relationship-building) linked to brand evangelism through dual mediating pathways: brand image (symbolic‌‌ pathway) and customer trust (relational pathway). Loyalty program participation moderates the trust–evangelism link (H7), and visit frequency moderates the brand image–evangelism link (H8). Perceived CSR is included as a control variable. Hypotheses are tested separately for Vietnam and Thailand using PLS-SEM with multi-group analysis. **Source: Authors’ own work.**

The proposed research model is illustrated in Fig 1.

## 3. Methodology

### 3.1. Research context

This study was conducted in Vietnam and Thailand, two leading hospitality markets within ASEAN that are experiencing rapid growth in both domestic and international tourism. Vietnam has positioned cities such as Ho Chi Minh City, Hanoi, and Da Nang as hubs of business and leisure travel, characterized by strong adoption of digital technologies in hospitality services [18]. Thailand, represented here by Bangkok and Chiang Mai, has long been recognized for its hospitality industry rooted in cultural heritage and service orientation [19]. These two countries were deliberately selected because they combine similarities rapidly expanding upscale hotel sectors with meaningful contrasts in cultural norms, service traditions, and digital adoption levels. This makes them theoretically appropriate for examining whether the mechanisms leading to brand evangelism are culturally invariant or contingent. By focusing on four- and five-star hotels, the study targeted service contexts where digital competence, proactive assistance, and relationship-building are most salient, as these hotels employ trained staff and integrate advanced digital systems to deliver personalized experiences. Hotel selection within each city followed a purposive approach. In Vietnam, hotels were identified from the official certification registry of the Vietnam National Authority of Tourism, restricting inclusion to properties holding current 4- or 5-star ratings. In Thailand, the Thailand Hotel Association’s registry served as the sampling frame. Within each city, a minimum of three hotels were recruited to represent both international chain affiliates and locally owned boutique properties, capturing variation in service culture and digital infrastructure. Hotel managers were contacted directly to obtain formal permission and to facilitate researcher presence in lobbies and reception areas during data collection periods. This research context thus provides both practical relevance and theoretical leverage for testing the generalizability of the proposed model.

### 3.2. Sample and data collection

Data were collected in Vietnam and Thailand, two leading ASEAN hospitality markets with rapid growth and distinct cultural norms. In Vietnam, surveys were conducted in Ho Chi Minh City, Hanoi, and Da Nang; in Thailand, data collection took place in Bangkok and Chiang Mai. These sites captured both business and leisure travellers. A full profile of the sample characteristics is provided in Table 2.

**Table 2:** Sample profile of respondents.

Characteristics	Vietnam (n = 236)	Thailand (n = 192)	Total (N = 428)
Age (years)	Mean = 31.16	Mean = 37.62	Mean = 33.97
Gender			
Male	80 (34.0%)	56 (29.2%)	136 (31.8%)
Female	156 (66.0%)	136 (70.8%)	292 (68.2%)
Minimum Stay Requirement	≥ 2 nights in 4–5 star hotel	≥ 2 nights in 4–5 star hotel	Applied consistently
Recruitment Method	On-site survey, Wave 1; online follow-up, Wave 2	On-site survey, Wave 1; online follow-up, Wave 2	Two-wave design
Screening and Data Cleaning	14 cases removed (inattentive patterns, response time checks)	11 cases removed (inattentive patterns, response time checks)	25 cases removed total
Final Valid Responses	236	192	428
Effective Response Rate	62.913%	–	–

Percentages are based on valid responses. Response rate calculated as usable cases divided by total recruited across two waves.

Source: Authors’ own work.

A two-wave, time-lagged design with a four-week interval was used to reduce common method variance [24]. This interval balances bias reduction with attrition concerns: short lags of 2–4 weeks sustain engagement [24,25], while longer lags risk dropout. Consistent with hospitality research practice, four weeks was adopted as a rigorous compromise [6,15].

In Wave 1, trained assistants recruited eligible guests (minimum two-night stay) in hotel lobbies (Vietnam) and via manager-supported invitations (Thailand). After screening, participants completed the first survey on-site (paper, tablet, or QR code). Four weeks later, the online follow-up measured brand evangelism, loyalty program participation, and visit frequency. Participation was encouraged by hotel collaboration, assurances of anonymity, and modest incentives equivalent to USD 5 (MoMo/ZaloPay top-up in Vietnam; mobile card or voucher in Thailand).

The study followed ethical guidelines and was approved by the Institutional Review Board (IRB2024-0103-017). The recruitment period began on 05 March 2024 and concluded on 28 June 2024. Data collection followed a two-wave design, with a four-week interval separating the first and second waves. All participants were adults aged 18 years or older. Informed consent was obtained prior to participation through an electronic confirmation step, whereby individuals were required to indicate consent via a mandatory checkbox before accessing the survey in both waves of data collection. No minors participated in the study, and parental consent was therefore not required. No waiver of informed consent was sought or granted. Consent was documented automatically by the survey platform through its built-in response metadata.

The final dataset comprised 428 valid responses: 236 from Vietnam and 192 from Thailand, with a 62.9% effective response rate. Demographic heterogeneity (mean age = 31.16 in Vietnam vs. 37.62 in Thailand; gender slightly female-skewed) strengthens generalisability (Mansoor *et al*., 2025). An a priori G*Power analysis (α = .05, power = .95, f² = .06) indicated a minimum sample of ≈260; the achieved N = 428 far exceeded this threshold, affording > .99 post hoc power for such effects.

### 3.3. Measures

All constructs were measured using established multi-item scales adapted from prior research to ensure content validity. Items were drawn from sources widely cited in hospitality and marketing research, with contextual adjustments to the hotel setting. Responses were recorded on a seven-point Likert scale ranging from 1 = strongly disagree to 7 = strongly agree.

Frontline employees’ digital competence was measured with a five-item scale from [26]. Proactive assistance was assessed using four items from [27], while relationship-building was measured with four items from [7]. Brand image was evaluated using a five-item instrument by [11]. Customer trust was assessed with a four-item adaptation of [13] scale, and brand evangelism with a four-item scale from [28].

Perceived corporate social responsibility (CSR) was included as a control variable, using an eight-item scale from Swaen *et al*. (2021) to account for potential confounding effects. Moderators included loyalty program participation (binary: 1 = participant, 0 = non-participant) and visit frequency (categorical: 1 = first-time, 2 = occasional [2–3 stays], 3 = frequent [4 + stays]). These measures ensured perceptual and relational pathways were captured, consistent with recommendations for construct operationalisation in hospitality research [2].

### 3.4. Data analysis

The structural model was analysed using partial least squares structural equation modelling (PLS-SEM) with SmartPLS 4, recommended for complex models with mediation and moderation [29]. PLS-SEM was selected over covariance-based SEM (CB-SEM) for several substantive reasons. CB-SEM is designed for confirmatory testing of well-established theoretical structures with large, normally distributed samples; it performs poorly when models are exploratory, include complex path configurations, or when group sample sizes are asymmetric. In the present study, the cross-national extension of a nascent evangelism framework represents a theory-building rather than strictly confirmatory aim, sub-group sample sizes are unequal (236 vs. 192), and Mardia’s test indicated departure from multivariate normality. PLS-SEM was therefore appropriate as it is robust against non-normal data, appropriate for models with multiple constructs and modest samples (236 in Vietnam, 192 in Thailand; N = 428), and offers both explanatory and predictive relevance. Reliability and validity were confirmed through Cronbach’s alpha, composite reliability, and average variance extracted, all exceeding recommended thresholds [29]. Discriminant validity was established using HTMT ratios (<0.850), with all loadings above 0.7. Mediation was tested via bootstrapping with 5,000 resamples and bias-corrected confidence intervals [25]. Moderation was assessed using multi-group analysis (MGA) for loyalty program participation and a product-indicator approach for visit frequency. Common method bias (CMB) was minimal, with a one-factor test showing 22.062% variance explained (<50%; [24,30]) and collinearity checks showing VIF < 3.3 (Kock, 2015). Measurement invariance across Vietnam and Thailand was confirmed using MICOM [31], with configural, compositional, and equality of means and variances established, enhancing credibility and replicability [2,6]. Specifically, following the three-step procedure recommended by Henseler et al. [31] and elaborated by Gannon et al. (2021): Step 1 (configural invariance) confirmed the model converged properly in both groups with identical indicator assignments; Step 2 (compositional invariance) used permutation tests with 5,000 draws and confirmed all composite correlations exceeded 0.90, validating cross-national construct equivalence; Step 3 confirmed equality of composite means and variances, permitting valid group-level path comparisons.

## 4. Results

### 4.1. Measurement model

The measurement model was assessed separately for Vietnam and Thailand to establish reliability and validity. For Vietnam, Cronbach’s alpha values ranged from 0.846 to 0.917, composite reliability (CR) from 0.889 to 0.941, and average variance extracted (AVE) from 0.607 to 0.800. For Thailand, Cronbach’s alpha ranged from 0.873 to 0.926, CR from 0.913 to 0.939, and AVE from 0.639 to 0.793. All coefficients exceeded recommended thresholds (α > 0.70, CR > 0.70, AVE > 0.50; [29]). Outer loadings exceeded 0.70 for all indicators, confirming item reliability, and VIF values (1.68–3.30 in Vietnam; 1.74–3.27 in Thailand) remained well below the cut-off of 5, ruling out multicollinearity concerns (Table 3).

**Table 3:** Construct measurement validation.

Construct	Item	Outer loading		VIF		Cronbach’s α	CR	AVE
		VN	TH	VN	TH	VN / TH	VN / TH	VN / TH
Digital Competence (DC)	DC1. Employees effectively use digital tools	0.747	0.838	1.838	2.794	0.846 / 0.905	0.889 / 0.929	0.617 / 0.725
	DC2. Employees are proficient in digital platforms	0.815	0.881	2.312	3.272			
	DC3. Employees resolve issues via digital systems	0.721	0.842	1.704	2.417			
	DC4. Employees integrate digital tools for personalisation	0.811	0.842	1.681	2.469			
	DC5. Employees’ digital skills inspire confidence	0.828	0.852	2.067	2.415			
Proactive Assistance (PA)	PA1. Anticipate needs before I ask	0.875	0.850	2.707	2.171	0.897 / 0.873	0.928 / 0.913	0.764 / 0.725
	PA2. Notice potential problems early	0.835	0.858	2.013	2.375			
	PA3. Provide solutions in advance	0.876	0.812	2.605	1.744			
	PA4. Proactively offer help and guidance	0.908	0.883	3.263	2.714			
Relationship-Building (RB)	RB1. Build long-term bonds	0.712	0.795	1.521	2.347	0.830 / 0.891	0.878 / 0.920	0.644 / 0.744
	RB2. Remember preferences from prior visits	0.826	0.869	1.978	2.490			
	RB3. Show genuine interest in me	0.761	0.889	1.948	2.545			
	RB4. Invest effort to maintain relationship	0.899	0.893	1.799	2.295			
Brand Image (BI)	BI1. Strong, favourable brand image	0.890	0.887	3.135	3.027	0.920 / 0.922	0.940 / 0.941	0.758 / 0.763
	BI2. Considered prestigious	0.855	0.855	2.525	2.614			
	BI3. Well respected versus competitors	0.866	0.866	2.550	2.559			
	BI4. Consistent with high standards	0.864	0.885	2.698	3.017			
	BI5. Leaves lasting positive impression	0.879	0.873	2.988	2.750			
Customer Trust (CT)	CT1. Trust brand to keep promises	0.892	0.909	2.722	3.197	0.917 / 0.912	0.941 / 0.938	0.800 / 0.791
	CT2. Brand is honest	0.883	0.897	2.824	2.960			
	CT3. Trust brand to act in my best interest	0.906	0.894	3.300	2.841			
	CT4. Confidence in relying on brand	0.895	0.855	2.848	2.382			
Brand Evangelism (BE)	BE1. Actively recommend brand	0.881	0.874	2.740	2.612	0.905 / 0.913	0.934 / 0.939	0.779 / 0.793
	BE2. Say positive things	0.862	0.903	2.213	3.138			
	BE3. Encourage family/friends	0.890	0.908	2.935	3.097			
	BE4. Defend brand from criticism	0.898	0.877	2.951	2.623			
Perceived CSR	CSR1. Hotel is socially responsible	0.788	0.799	2.601	3.004	0.915 / 0.926	0.925 / 0.934	0.607 / 0.639
	CSR2. Respects environment	0.785	0.785	2.796	2.382			
	CSR3. Contributes to community	0.757	0.806	2.583	1.910			
	CSR4. Treats employees fairly	0.851	0.775	2.434	2.461			
	CSR5. Transparent operations	0.873	0.893	2.303	2.958			
	CSR6. Communicates responsibilities clearly	0.716	0.702	2.085	2.440			
	CSR7. Balances profit with responsibility	0.728	0.799	2.063	2.271			
	CSR8. Demonstrates CSR overall	0.718	0.825	1.982	2.453			

VN = Vietnam; TH = Thailand; CR = composite reliability; AVE = average variance extracted. Country labels are shown in the column header row rather than repeated within each cell. VIF values are separated into country-specific sub-columns to reduce horizontal crowding. All outer loadings exceed 0.70, and all VIF values remain below 5.

Source: Authors’ own work.

Discriminant validity was verified using the heterotrait–monotrait ratio of correlations (HTMT). In Vietnam, HTMT ratios ranged from 0.053 to 0.627, and in Thailand from 0.110 to 0.726, all below the conservative 0.85 cut-off [31]. Cross-national contrasts were evident: in Thailand, stronger associations emerged between customer trust and brand evangelism (HTMT = 0.726) and between customer trust and relationship-building (HTMT = 0.762), underscoring the relational emphasis of a trust-driven service culture. By contrast, Vietnamese customers emphasised symbolic differentiation, with brand image showing stronger ties to brand evangelism (HTMT = 0.627) (Table 4).

**Table 4:** Discriminant validity (HTMT ratios).

Constructs	DC	PA	RB	BI	CT	BE
Panel A. Vietnam						
DC	–					
PA	0.311	–				
RB	0.100	0.055	–			
BI	0.286	0.342	0.093	–		
CT	0.289	0.446	0.053	0.392	–	
BE	0.283	0.311	0.079	0.627	0.416	–
Panel B. Thailand						
DC	–					
PA	0.371	–				
RB	0.113	0.113	–			
BI	0.365	0.252	0.125	–		
CT	0.350	0.233	0.110	0.555	–	
BE	0.358	0.371	0.155	0.665	0.726	–

HTMT = heterotrait–monotrait ratio. All HTMT values are below the conservative threshold of 0.85, supporting discriminant validity.

Source: Authors’ own work.

### 4.2. Structural model

The structural model was estimated using bootstrapping with 5,000 subsamples. Frontline employee attributes significantly predicted brand evangelism in both countries, with one exception. Digital competence was positive in Vietnam (β = 0.301, t = 5.108, p < .001) and Thailand (β = 0.241, t = 5.018, p < .001), supporting H1a (Table 5). Proactive assistance was significant in both Vietnam (β = 0.333, t = 5.477, p < .001) and Thailand (β = 0.163, t = 3.473, p < .001), supporting H1b. Relationship-building did not reach significance in Vietnam (β = 0.092, n.s.) but was weakly positive in Thailand (β = 0.084, p < .05), yielding partial support for H1c. Brand image (VN: β = 0.458, p < .001; TH: β = 0.355, p < .001) and trust (VN: β = 0.248, p < .01; TH: β = 0.440, p < .001) further enhanced evangelism, confirming H4–H5.

**Table 5:** Structural model results.

Hypothesis / Path	Vietnam (β, t, p)	Thailand (β, t, p)	Supported		
**Panel A. Direct effects on brand evangelism**					
H1a: DC → BE	0.301, 5.108, ***	0.241, 5.018, ***	Yes		
H1b: PA → BE	0.333, 5.477, ***	0.163, 3.473, ***	Yes		
H1c: RB → BE	0.092, 1.100, n.s.	0.084, 1.701, *	No (VN) / Yes (TH)		
H4: BI → BE	0.458, 7.265, ***	0.355, 6.099, ***	Yes		
H5: CT → BE	0.248, 2.926, **	0.440, 5.783, ***	Yes		
**Panel B. Direct effects on brand image**					
H2a: DC → BI	0.281, 4.886, ***	0.309, 5.025, ***	Yes		
H2b: PA → BI	0.321, 5.273, ***	0.217, 3.166, **	Yes		
H2c: RB → BI	0.111, 1.397, n.s.	0.112, 1.567, n.s.	No		
**Panel C. Direct effects on customer trust**					
H3a: DC → CT	0.263, 4.538, ***	0.299, 4.968, ***	Yes		
H3b: PA → CT	0.416, 8.419, ***	0.196, 2.816, **	Yes		
H3c: RB → CT	−0.009, 0.126, n.s.	0.100, 1.356, n.s.	No		
**Panel D. Indirect effects: mediation via brand image (H6a)**					
**Indirect path**	**Vietnam (β, t, p)**	**Thailand (β, t, p)**	**VAF VN**	**VAF TH**	**Supported**
DC → BI → BE	0.129, 4.276, ***	0.110, 3.653, ***	42.70%	39.40%	Yes
PA → BI → BE	0.147, 4.440, ***	0.077, 2.751, **	44.20%	22.50%	Yes
RB → BI → BE	0.051, 1.358, n.s.	0.040, 1.528, n.s.	55.50%	26.50%	No
**Panel E. Indirect effects: mediation via customer trust (H6b)**					
**Indirect path**	**Vietnam (β, t, p)**	**Thailand (β, t, p)**	**VAF VN**	**VAF TH**	**Supported**
DC → CT → BE	0.065, 2.470, **	0.131, 3.872, ***	21.60%	47.10%	Yes
PA → CT → BE	0.103, 2.685, **	0.086, 2.397, **	31.00%	25.30%	Yes
RB → CT → BE	−0.002, 0.120, n.s.	0.044, 1.275, n.s.	−2.4%	29.50%	No
**Panel F. Moderation effects**					
**Moderation path**	**Vietnam**	**Thailand**	**Supported**		
H7: Loyalty programme participation on CT → BE	L = 1: 0.034, 0.377, n.s.L = 0: 0.248, 2.926, **	L = 1: 0.404, 5.661, ***L = 0: 0.440, 5.783, ***	No (VN) / Yes (TH)		
H8: Visit frequency on BI → BE	+1 SD: 0.433, 4.919, *** − 1 SD: 0.483, 6.171, ***	+1 SD: 0.229, 3.130, ** − 1 SD: 0.481, 6.489, ***	Yes (both; differing patterns)		
**Panel G. Cross-national comparisons (PLS-MGA)**					
**Path difference**	**Result**				
CT → BE (Δβ = −0.192)	Significant (p = .044)				
PA → CT (Δβ = 0.219)	Significant (p = .005)				
**Panel H. Explained variance**					
**Endogenous construct**	**Vietnam R² (Adj. R²)**	**Thailand R² (Adj. R²)**			
BE	0.400 (0.373)	0.603 (0.581)			
BI	0.185 (0.174)	0.168 (0.154)			
CT	0.235 (0.225)	0.149 (0.136)			

BE = brand evangelism; BI = brand image; CT = customer trust; DC = digital competence; PA = proactive assistance; RB = relationship-building; VAF = variance accounted for; PLS-MGA = partial least squares multi-group analysis. **Source:** Authors’ own work.

Antecedents of brand image and trust were largely supported. For image, DC and PA were significant in both countries, while RB was not. For trust, DC and PA were again significant, whereas RB remained non-significant.

Mediation tests confirmed dual mechanisms. Brand image mediated the effects of DC and PA in both countries (VN VAF = 42.7–44.2%; TH VAF = 39.4%–22.5%), but not RB. Trust also mediated DC and PA (VN VAF = 21.6–31.0%; TH VAF = 47.1%–25.3%), but not RB. Overall, Vietnam relied more on image-based mediation, while Thailand leaned on trust-based pathways.

**Fig 2:**
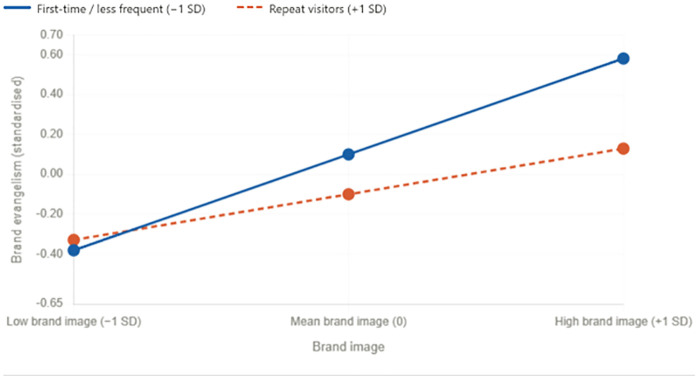
Moderation effects: interaction plot of brand image × visit frequency on brand evangelism. Source: Authors own work.

Moderation results revealed cultural contingencies. Loyalty program participation did not affect Vietnam but strengthened the trust–evangelism link in Thailand, partially supporting H7. Visit frequency moderated the image–evangelism path in both markets, though with differing slopes (see Fig 2): effects were consistent in Vietnam but diverged in Thailand, where first-time and less frequent visitors showed a stronger brand image–evangelism effect (β = 0.481 at −1 SD) compared to repeat visitors (β = 0.229 at +1 SD), indicating that the symbolic power of brand image attenuates rather than compounds with visit accumulation in Thailand.

Cross-national comparisons (PLS-MGA) identified two significant differences: CT → BE was stronger in Thailand (Δβ = −0.192, p = .044), whereas PA → CT was stronger in Vietnam (Δβ = 0.219, p = .005). All other paths were stable across contexts, supporting broad model consistency.

Explained variance was substantial. In Vietnam, R^2^ was 0.400 for evangelism, 0.185 for image, and 0.235 for trust. In Thailand, R^2^ was higher for evangelism (0.603) but lower for image (0.168) and trust (0.149). Model fit was adequate, with SRMR values below 0.08 in both samples (VN = 0.050; TH = 0.049). These results validate the model, confirm its explanatory strength, and highlight meaningful symbolic–relational contrasts between Vietnam and Thailand.

## 5. Discussion

### 5.1. Discussion of findings

The findings strongly support the dual-mediator framework, confirming that both brand image and trust channel frontline employee behaviours into evangelism. Extending earlier work that highlighted symbolic mechanisms, the results demonstrate that trust is equally indispensable. Evangelism is not simply admiration but a high-commitment act where customers risk their own credibility; both symbolic alignment and relational assurance are therefore required.

Cross-national analysis underscores cultural contingencies. In Vietnam, brand image emerged as the stronger mediator (VAF 42.7–44.2%), suggesting that evangelism functions as status-linked signalling where alignment with symbolic reputation enhances identity affirmation. In contrast, in Thailand trust accounted for larger variance (VAF up to 47.1%), reflecting the primacy of relational credibility and harmony in high-context cultures. These differences confirm that evangelism mechanisms are culturally contingent: symbolic admiration dominates where reputation is paramount, while trust prevails where dependability and harmony are central.

Boundary conditions refined these insights. Loyalty program participation strengthened the trust–evangelism path in Thailand but not in Vietnam, indicating that institutional scaffolding amplifies relational cues only in relationally oriented contexts. Visit frequency moderated the brand image–evangelism link in both countries but with differing patterns: Vietnamese customers responded consistently across visit frequencies, whereas Thai customers’ responses diverged more sharply, with first-time and less frequent visitors exhibiting a notably stronger brand image–evangelism effect (β = 0.481 at −1 SD) compared to repeat visitors (β = 0.229 at +1 SD). This counterintuitive pattern suggests that in Thailand, accumulated visits may erode rather than reinforce image-based advocacy, possibly because repeat exposure elevates expectations and exposes service inconsistencies that undermine the symbolic value of the brand. These findings highlight that contextual reinforcements such as loyalty systems and accumulated encounters condition how symbolic and relational signals translate into evangelism.

### 5.2. Theoretical contributions

This study reconceptualises brand evangelism with two core implications. First, it positions advocacy as a trust-dependent behaviour, thereby anchoring evangelism research more firmly within relationship marketing and signalling theory [7,13]. While prior work has tended to emphasise symbolic drivers, the present findings demonstrate that trust is a parallel mediator of equal weight. Earlier studies may have overstated the symbolic pathway precisely because they omitted trust from their models [11,12]. Evangelism thus emerges at the intersection of reputational admiration and relational credibility, with cultural context shaping which mechanism dominates [28].

Second, the study extends social identity theory by showing that brand identification is culturally contingent. In Vietnam, the dominant mediating role of brand image (VAF = 42.7–44.2%) indicates that evangelism functions as a form of symbolic signalling, where reputation alignment enhances identity. In Thailand, by contrast, trust exerts stronger mediating effects (VAF up to 47.1%), demonstrating that relational credibility and dependability carry greater weight in high-context cultures [9,32]. These contrasts caution against assuming cross-national homogeneity in evangelism and reinforce recent calls to integrate cultural affordances into evangelism theory [4].

Boundary conditions further refine this framework. Loyalty program participation amplified the trust–evangelism path in Thailand but was non-significant in Vietnam, suggesting that structured relational scaffolds strengthen advocacy only where cultural norms already privilege relational credibility [10]. Visit frequency moderated the brand image–evangelism link in both countries, but with different slopes: consistent symbolic effects in Vietnam contrasted with sharper divergence among Thai customers, where first-time and less frequent visitors showed a stronger brand image–evangelism effect (β = 0.481 at −1 SD) while repeat visitors exhibited a notably attenuated effect (β = 0.229 at +1 SD). This asymmetry underscores that evangelism is contingent on structural and experiential contexts [23].

The counterintuitive finding that repeated visits attenuate rather than amplify image-based brand evangelism in Thailand challenges linear reinforcement assumptions. Prior research has often treated frequency as a straightforward trust enhancer [16,33]. Yet the present results show that fragility arises from service variability, unmet expectations, and cultural norms of indirect dissatisfaction, particularly in high-context settings.

Together, these dynamics extend the boundaries of relationship marketing by portraying evangelism as a dynamic, expectation-laden process rather than a guaranteed outcome of relational depth. From a managerial perspective, hotels must invest not only in relationship-building but also in consistency management, expectation calibration, and culturally sensitive feedback systems. Monitoring repeat-guest expectations and minimising variability across encounters are crucial to reducing trust fragility and converting long-term patronage into sustained advocacy [2,6].

### 5.3. Practical implications

The findings provide strategic insights for hospitality leaders in ASEAN. In Vietnam, brand image was the stronger mediator of evangelism, highlighting the centrality of symbolic differentiation in a modernising service economy. Digital competence and proactive assistance significantly shaped both image and trust, suggesting that managers should treat digitalisation not merely as an operational enhancement but as a strategic lever for building symbolic value propositions. Seamless platforms, automation, and consistent image management can position hotels as technologically credible while also reinforcing status-linked identity cues.

Concretely, hotels in Vietnam should invest in staff training programs that emphasize digital tool proficiency such as mobile check-in systems, AI-assisted guest personalization, and real-time preference tracking as these competencies directly enhance brand image perceptions (β = 0.281) and subsequently drive evangelism (β = 0.458). Front-desk and concierge staff should be equipped with tablet-based guest history dashboards to enable seamless proactive service that signals both technical sophistication and genuine attention. Regular brand perception audits conducted quarterly and tied to guest satisfaction scores can help managers identify gaps between intended brand image and guest perceptions before these gaps erode advocacy intent.

In Thailand, trust emerged as the dominant mediator, with customer evangelism more dependent on relational credibility than symbolic alignment. Here, service innovation should prioritise transparency, dependability, and continuity. High-touch practices such as culturally attuned personalisation, consistent delivery, and relational protocols are particularly effective in sustaining harmony and reliability, reflecting the stronger CT → BE pathway observed in the Thai sample.

In practical terms, Thai hotel managers should prioritise building interpersonal consistency protocols: for instance, assigning dedicated relationship managers to repeat guests, maintaining digital guest preference records that are shared across all service touchpoints, and implementing structured post-stay follow-up communications that reinforce the sense of ongoing relational investment. The significant loyalty program – trust – evangelism chain in Thailand (H7 supported: β = 0.404, p < .001 for loyalty members) suggests that expanding and communicating loyalty tier benefits such as guaranteed room upgrades, priority service access, and personalised milestone recognition can tangibly increase advocacy rates. Managers should also heed the moderation finding that first-time visitors in Thailand showed stronger image–evangelism sensitivity (β = 0.481 at −1 SD vs. 0.229 for repeat visitors), meaning that first impressions are particularly high-stakes and warrant heightened investment in symbolic onboarding experiences such as curated welcome rituals and culturally resonant arrival protocols.

Loyalty programs and visit frequency also demonstrated culturally specific boundary conditions. In Vietnam, loyalty effects were negligible, consistent with an image-dominant pathway where symbolic cues are already sufficient to trigger advocacy. In Thailand, however, loyalty participation significantly reinforced the trust–evangelism link, underscoring that institutional scaffolding can amplify relational assurance. Visit frequency moderated the brand image–evangelism link in both markets but showed sharper divergence in Thailand, where repeat encounters intensified advocacy but also risked fragility if expectations were not met.

For cross-border hotel groups, these results emphasise the need for regional agility. Uniform service models risk cultural dissonance and reduced advocacy. Instead, firms should align digital transformation with Vietnam’s symbolic logic while anchoring Thai operations in relational strategies. This dual approach leverages cultural heterogeneity to strengthen competitive positioning, build sustained loyalty, and maximise returns on innovation.

## 6. Conclusion, limitations and future research

### 6.1. Conclusion

This study advances prior work by introducing brand image and customer trust as dual mediators, integrating loyalty program participation and visit frequency as boundary conditions, and validating the model across two ASEAN markets. The findings demonstrate that Vietnamese customers respond more strongly to symbolic cues conveyed through brand image, whereas Thai customers place greater weight on trust, reflecting cultural contingencies in how frontline employee attributes shape evangelism. These insights refine relationship marketing and social identity theory by clarifying the dual pathways through which advocacy emerges and by revealing how structural and experiential contexts condition these effects. Beyond theoretical contributions, the study offers actionable guidance for hospitality managers: strategies in Vietnam should foreground symbolic differentiation and digital competence, while in Thailand they should prioritise trust-based continuity and relational assurance. Together, these results underscore the need to embed cultural variation into both theory and practice when designing service encounters that convert customer experiences into sustained advocacy.

### 6.2. Limitations and future research

This study is subject to several limitations that also provide opportunities for future research. First, the analysis was confined to Vietnam and Thailand, which limits the generalisability of the findings. Future studies could extend the model to other ASEAN economies or undertake cross-continental comparisons to examine whether the identified mechanisms hold across diverse service cultures. Second, the reliance on customer-only survey data offers only a partial view of service encounters. Incorporating employee and managerial perspectives through multi-source or mixed-method designs would provide a more comprehensive understanding of how frontline behaviours foster evangelism. Third, the study’s focus on luxury hotels narrows its applicability to high-end contexts. Testing the framework in budget and mid-tier hotels, as well as on digital-first platforms such as Agoda or Booking.com, would expand its boundary conditions and generate richer insights. Addressing these limitations will enhance the robustness and external validity of future research on brand evangelism in hospitality.
